# First report of the mitochondrial DNA sequences of the giant clam, *Tridacna gigas* (Tridacnidae Tridacna)

**DOI:** 10.1080/23802359.2020.1830729

**Published:** 2020-10-23

**Authors:** Haitao Ma, Zhiming Xiang, Yang Zhang, Jun Li, Yanping Qin, Yuehuan Zhang, Ziniu Yu

**Affiliations:** a Key Laboratory of Tropical Marine Bio-resources and Ecology, Guangdong Provincial Key Laboratory of Applied Marine Biology, South China Sea Institute of Oceanology, Chinese Academy of Sciences, Guangzhou, China; b South China Sea Bio-Resource Exploitation and Utilization Collaborative Innovation Center, Guangzhou, China; c Innovation Academy of South China Sea Ecology and Environmental Engineering, Chinese Academy of Sciences, Guangzhou, China

**Keywords:** *Tridacna gigas*, mitochondrial genome, phylogenetic relationship

## Abstract

In this study, we present the first complete mitochondrial genome sequence of the giant clam *Tridacna gigas*. The total length of the mitogenome is 19,558 bp. It contains the typical mitochondrial genomic structure, including 13 protein-coding genes, 23 transfer RNA genes, two ribosomal RNA genes, and one control region (D-loop). Mitogenome base composition is biased toward A + T content, at 57.6%. A phylogenetic tree based on complete mitogenome sequences revealed that, within the genus *Tridacna*, *T. gigas* is closely related to *T. derasa*.

Giant clams of the genus Tridacna (Cardiidae: Tridacnidae) are conspicuous and colorful bivalves that inhabit coral reefs across the Indo-Pacific. Among them, *Tridacna gigas* is the largest externally shelled living mollusk (Rosewater 1965). The abundance of the giant clam *T. gigas* in coral reef environments is declining, mainly due to overfishing (Lucas 1988), and the species is on the UN list of endangered species (Hilton-Taylor 2000). In this study, we sequenced the complete mitochondrial genome of *T. gigas* to further the study of the taxonomy and phylogenetic relationships of Tridacnidae by increasing the amount of available molecular data.

The specimen was collected from Sanya, Hainan province, China (N109.51, E18.21) by a local fisherman, and stored in Tropical Marine Biodiversity Collections of South China Sea (TMBC), Chinese Academy of Sciences, Guangzhou, China (specimen accession number: TMBC030712). The total genomic DNA was extracted following the modified CTAB DNA extraction protocol (Attitalla 2011), followed by library preps and pair-end sequencing (2 × 150 bp) with HiSeq (Illumina, San Diego, CA). Approximately, 5884 Mb of raw data and 5126 Mb of clean data were obtained, and de novo assembled by the SOAP de novo software (Zhao et al. 2011) with an average of approximately 280× coverage.

The mitogenome of *T. gigas* is 19,558 bp in length (GenBank accession number MT755623). It contains 13 protein-coding genes (PCGs), 23 transfer RNA genes (tRNAs), two ribosomal RNA (*12S rRNA* and *16S rRNA*) genes, and a non-coding control region (D-loop). The mitogenome base composition of *T. gigas* is biased toward A + T content at 57.6% (25.5% A, 32.1% T, 16.7% C, 25.7% G). The 13 identified PCGs vary in length from 114 to 1728 bp. COI, ATP8, ND4, COII, COIII, and Cytb initiate with TTG as the start codon; while ND2 and ND5 begin with GTG; ND4L and ND6 begin with ATG; ATP6 with ATT; ND3 with ATA and NDI with ATC. Five types of stop codons revealed are TAA (COIII, Cytb, ND4L, ATP6, ND3, ND1), TAG (COI, ATP8, COII, ND6), GTA (ND4), CTG (ND5), and TGT (ND2). The lengths of the 23 tRNA genes range from 62 to 71 bp, and all of the tRNA genes have typical secondary structure. The *12S rRNA* gene is located between tRNA-Leu and ND6, and is 928 bp long, while the *16S rRNA* gene is located between tRNA-Ile and ND1, with a length of 1042 bp. A 3009 bp control region (D-loop) was located between tRNA-Arg and COII, with an A + T content of 51.55%.

A neighbor-joining phylogenetic tree of *T. gigas* and five other closely related species was constructed with the complete mitochondrial genomes using MEGA6 (Tamura et al. 2013) (Figure 1). The result showed that, within the genus Tridacna, *T. gigas* is closely related to *Tridacna derasa*.

**Figure 1.**
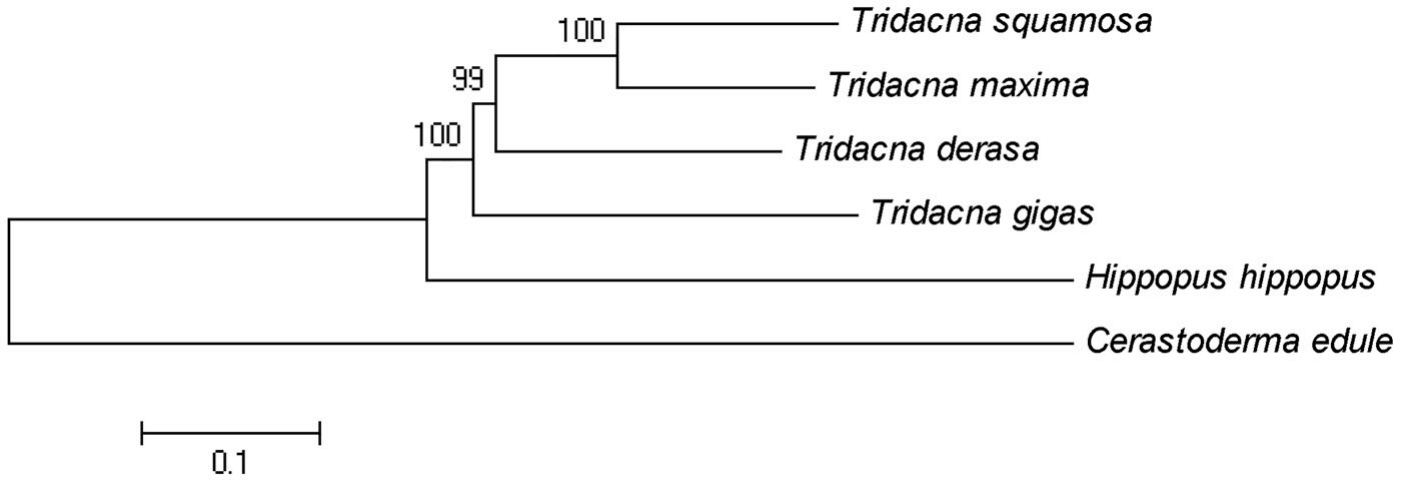
Neighbor-joining phylogenetic tree of *Tridacna gigas* and five other closely related species based on the complete mitochondrial genomes. GenBank accession numbers: *Cerastoderma edule* (MF374632); *Hippopus hippopus* (MG722975); *Tridacna derasa* (MG755811); *Tridacna maxima* (MK105973); *Tridacna squamosa* (KP205428).

## Data Availability

The authors confirm that the data supporting the findings of this study are available within the article and its supplementary materials. https://www.ncbi.nlm.nih.gov/nuccore/MT755623
